# Aging synaptic mitochondria exhibit dynamic proteomic changes while maintaining bioenergetic function

**DOI:** 10.18632/aging.100657

**Published:** 2014-05-05

**Authors:** Kelly L. Stauch, Phillip R. Purnell, Howard S. Fox

**Affiliations:** Department of Pharmacology and Experimental Neuroscience, University of Nebraska Medical Center, Omaha, NE 68198-5800, USA

**Keywords:** synaptic mitochondria, aging, proteomics, bioenergetics, hormesis

## Abstract

Aging correlates with a progressive impairment of mitochondrial homeostasis and is an influential factor for several forms of neurodegeneration. However, the mechanisms underlying age-related alterations in synaptosomal mitochondria, a neuronal mitochondria population highly susceptible to insults and critical for brain function, remain incompletely understood. Therefore this study investigates the synaptic mitochondrial proteomic and bioenergetic alterations that occur with age. The utilization of a state of the art quantitative proteomics approach allowed for the comparison of protein expression levels in synaptic mitochondria isolated from 5 (mature), 12 (old), and 24 (aged) month old mice. During the process of aging we find that dynamic proteomic alterations occur in synaptic mitochondria. Despite direct (mitochondrial DNA deletions) and indirect (increased antioxidant protein levels) signs of mitochondrial damage in the aged mice, there was an overall maintenance of mitochondrial function. Therefore the synaptic mitochondrial proteomic changes that occur with aging correlate with preservation of synaptic mitochondrial function.

## INTRODUCTION

Aging is a complex and heterogeneous process characterized by a progressive decline in physiologic function, followed by dysfunction and ultimately death. Aging is a major risk factor for most prevalent human diseases including several forms of neurodegeneration. Mitochondrial dysfunction, which has been implicated as a central player in aging theories for several decades, is among the nine recently characterized hallmarks of aging [1]. Dysfunctional mitochondria in aged mammals are described to exhibit a diminished capacity for ATP production, decreased membrane potential, and fragility [2]. Further, the presence of age-associated alterations and damage to mitochondrial DNA (mtDNA), proteins, and membrane lipids, which has also been extensively reported, may contribute to mitochondrial defects [3]. The cell has several mechanisms to prevent the accumulation of dysfunctional mitochondria, including mitochondrial fission and fusion cycles, biogenesis, and the selective elimination of damaged mitochondria by autophagy (termed mitophagy). However, disruptions in these processes have also been implicated in the aging process. The combination of damage and reduced ability to remove damaged mitochondria likely play a synergistic role in the aging process.

Neurons are highly dependent on the energy provided by mitochondrial respiration. The relationship between aging and mitochondrial function in neurons remains unclear. Because of their unique function and bioenergetic requirements, neurons have an uneven cellular mitochondria distribution. Areas with high energetic demand contain correspondingly higher density of mitochondria than the rest of the cell [4]. In particular, the synapse relies on high ATP levels for maintenance of membrane potential and vesicle mobilization [5]. In addition to their role in ATP production, neuronal mitochondria are necessary for maintaining proper Ca^2+^ homeostasis in the synapse, critical for the regulation of neurotransmission [6]. Most of the mitochondria that exist in the synapse are likely transported from the soma along the intricate neuronal microtubule system and dysregulation of this transport is associated with neurodegeneration. In addition to transport, proper mitochondrial localization depends on the intricate balance between mitochondrial fission and fusion proteins [7]. In order to better understand the mitochondrial changes that occur with age in the brain we focused on synaptic mitochondria, as they are uniquely positioned to suffer the effects of mitochondrial abnormalities with potentially critical impact on crucial brain functions.

Mitochondria display diversity in their proteomes, which allows each tissue to meet specialized functions [8]. With the specific need of the synapse for proper mitochondrial function, we hypothesized that alterations in the synaptic mitochondrial proteome are occurring with age. Although several studies have characterized the mouse brain synaptosomal proteome [9-11] and age-related proteomic changes in the brain [12, 13], the mitochondrial proteomic alterations that occur with age within the synaptosome remain uncharacterized. In this study, we performed a state of the art quantitative proteomic characterization of the aging mouse synaptic mitochondrial proteome. This approach allowed for the comparison of protein expression levels in synaptic mitochondria isolated from 5, 12, and 24 month old mice to identify the differentially expressed proteins. In combination with mitochondrial functional analysis, our results indicate that dynamic proteomic changes occur with aging to preserve synaptic mitochondrial function.

## RESULTS

### Quantitative analysis of the synaptic mitochondrial proteomic alterations during aging

**Figure 1:**
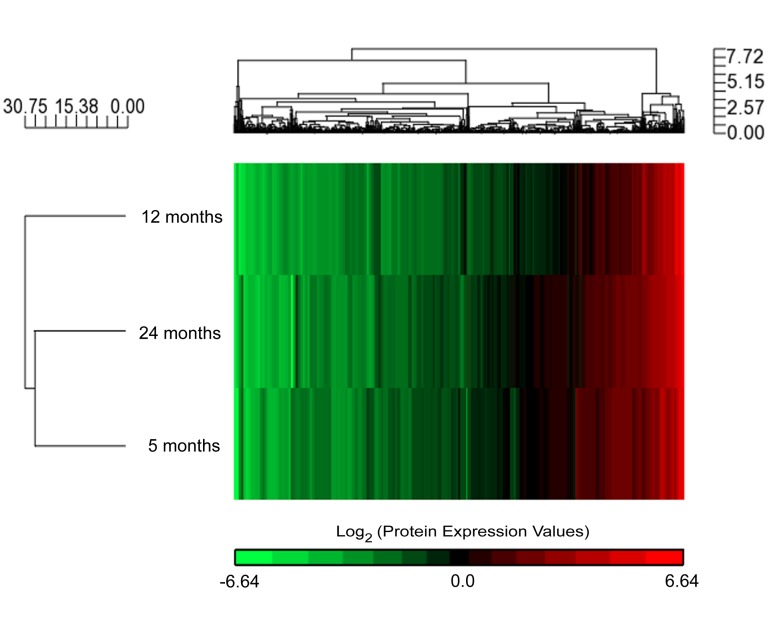
Aging associated synaptic mitochondrial proteomic changes Expression data derived from super-SILAC (H:L) ratios of 5, 12, and 24 month old mouse synaptic mitochondrial proteins. Hierarchical clustering analysis of log2 expression values for the common 898 identified and quantified proteins is presented here as a dendrogram.

To accurately quantify differences in the synaptic mitochondrial proteome with aging, we isolated synaptic mitochondria from 5 (mature), 12 (old), and 24 (aged) month old male C57BL/6 mice. Synaptic mitochondria were prepared from mouse brain tissue using Percoll density gradient centrifugation followed by immunopurification. The purity of the isolated synaptic mitochondria was verified using electron microscopy and previously described ultrastructural criteria (Supplemental Figure 3) [14]. Stable isotope labeling with amino acids in cell culture (SILAC) is an optimal method for accurate quantitative proteomics and the use of SILAC has been expanded for tissue proteome quantification by using a mix of multiple SILAC-labeled cell lines as internal standards (super-SILAC) [15-17]. Therefore, we prepared the mouse brain mitochondria super-SILAC mix that we developed and determined to serve as an appropriate standard for mouse synaptic and non-synaptic mitochondrial quantitative proteomics [18]. The pure isolated synaptic mitochondria were lysed and the proteins were mixed in a 1:1 ratio with the mouse brain mitochondria super-SILAC mix prior to processing by the filter-aided sample preparation (FASP) method [19] and acquisition of data on a TTOF 5600. Analysis of the resulting LC-MS/MS files in ProteinPilot using the Paragon method [20] identified and quantified a total of 898 proteins common between 5, 12, and 24 month old mouse synaptic mitochondria. The complete list of these 898 quantified and identified proteins for the synaptic mitochondria super-SILAC experiments is provided in the supplementary file 1. Hierarchical clustering analysis of the 898 common proteins using the protein expression values obtained from the SILAC heavy-to-light (labeled-to-unlabeled) ratios shows the global proteomic differences between synaptic mitochondria from each of the three ages (Figure 1). Based on protein expression, the synaptic mitochondria from 5 and 24 month old mice cluster together and as depicted by the length of the dendrogram clusters, the synaptic mitochondria from 12 months are only slightly more dissimilar compared to synaptic mitochondria from 5 and 24 months (Figure 1). The list of the 898 common proteins was also analyzed with MitoMiner, a database of the mitochondrial proteome, which annotated 758 proteins (84.4% of all identified proteins) as mitochondrial. The complete MitoMiner annotation output is provided in the supplementary file 2. As with all annotation software, the database is constantly updated therefore proteins not currently identified may actually be mitochondrial. These results indicate that the synaptic mitochondrial proteomic profile changes in response to aging.

### Proteomics predicts age-associated changes in mitochondrial transcriptional regulation

**Figure 2:**
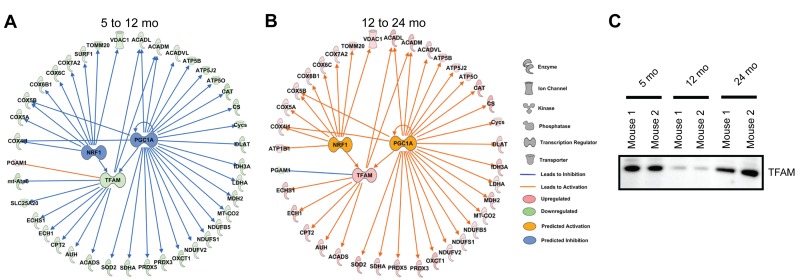
Analysis of quantitative proteomics predicts dynamic changes in mitochondrial transcriptional regulatory circuits with aging IPA generated NRF1, PGC1A, and TFAM upstream regulator networks overlaid with our proteomic expression data for synaptic mitochondria from (**A**) 5 to 12 month and (**B**) 12 to 24 month old mice. NRF1, PGC1A, and TFAM activity was predicted based on proteomic expression data. All proteins shown (except the nuclear proteins NRF1 and PGC1A) were found in the mitochondrial proteomic analysis. (**C**) Immunoblot orthogonal validation of TFAM protein expression.

In order to assess the factors guiding these changes, the mouse synaptic mitochondrial proteome changes during aging were evaluated using Ingenuity Pathway Analysis, which provides comprehensive pathway and network analysis tools for ‘omics data. The IPA Upstream Regulator and Downstream Effects Analysis enabled the prediction of regulators that may be driving the altered protein expression patterns seen. Therefore, we used this tool to investigate and visualize these regulators, and when overlaid with our proteomic expression data revealed dynamic changes in the synaptic mitochondrial pool with aging (Figure 2). Based on our proteomic expression data, the activity of two nuclear transcriptional regulators of genes encoding mitochondrial proteins, nuclear respiratory factor 1 (NRF1) and peroxisome proliferator-activated receptor gamma, coactivator 1 alpha (PGC1A) are predicted to change during aging. The activity of these transcription factors regulates the expression of the mitochondrial transcription factor A (TFAM), which is required for mtDNA maintenance and replication [21]. NRF1 and PGC1A are predicted to be inhibited during aging from 5 to 12 months, but activated from 12 to 24 months based on the synaptic mitochondrial proteome (Figure 2A and B). Consistent with the predicted inhibition and/or activation of NRF1 and PGC1A, IPA predicted changes in the expression of TFAM in aging synaptic mitochondria, which indeed was identified in our proteomic data (Figure 2A & B). The changes in TFAM protein expression during aging in synaptic mitochondria were validated orthogonally via immunoblotting (Figure 2C). These findings suggest that regulation of synaptic mitochondrial proteins during aging is not a linear process. Several targets of these transcriptional regulators are subunits of the electron transport chain complexes and antioxidant enzymes [22]; and changes in their activity affect mitochondrial biogenesis and function; therefore, we further investigated the processes of oxidative phosphorylation, response to reactive oxygen species (ROS), and mitochondrial dynamics.

### Quantitative proteomics reveals alterations in the expression of proteins involved in mitochondrial bioenergetics

**Figure 3:**
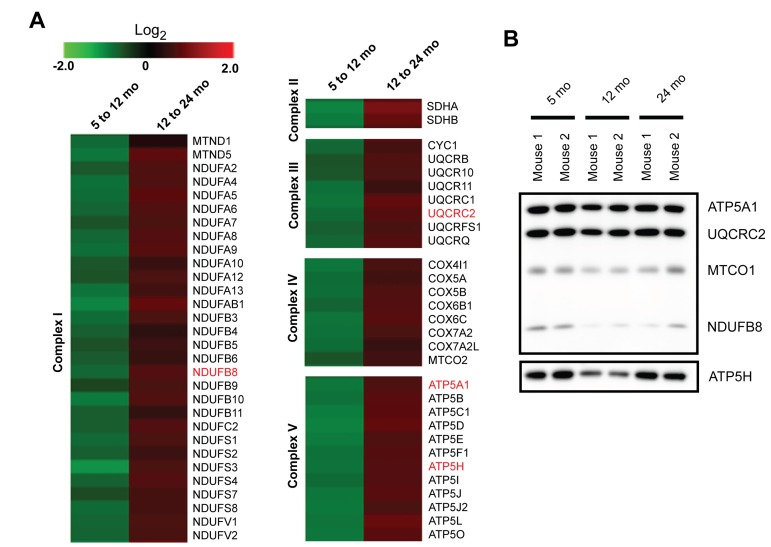
Quantitative proteomics reveals altered expression of protein subunits in the electron transport chain with aging (**A**) Heatmap of the protein expression changes from 5 to 12 months and 12 to 24 months in isolated synaptic mitochondria (log_2_ (12/5 months) and (24/12 months)). Proteins highlighted in red were verified orthogonally. (**B**) Immunoblot orthogonal validation of protein expression for selected proteins in (**A**).

Although our IPA upstream regulators analysis revealed alterations in several protein subunits of the ETC complexes, not all of the subunits are downstream targets of NRF1, PGC1A, or TFAM. Therefore, we decided to investigate the expression of the subunits of the protein complexes involved in the ETC and oxidative phosphorylation obtained from our proteomic experiments. Interestingly, all of the subunits of the ETC that we identified exhibit changes in protein expression during aging, specifically decreased expression from 5 to 12 months and increased expression from 12 to 24 months in synaptic mitochondria (Figure 3A). This result suggests dynamic changes in the mitochondrial proteome as a feedback or compensatory mechanism to manage any age-related decline of the ETC to ensure proper ATP production, which is especially important at the synapse. Immunoblotting was used to orthogonally validate the changes in expression of a subset of the ETC complex subunits (ATP5A1, UQCRC2, MTCO1, NDUFB8, and ATP5H) in the synaptic mitochondria during aging (Figure 3B). We next chose to address whether the overall complex subunit abundance correlates with the respiratory capacity of the synaptic mitochondria during aging.

### Functional bioenergetic analysis of aging synaptic mitochondria

**Figure 4:**
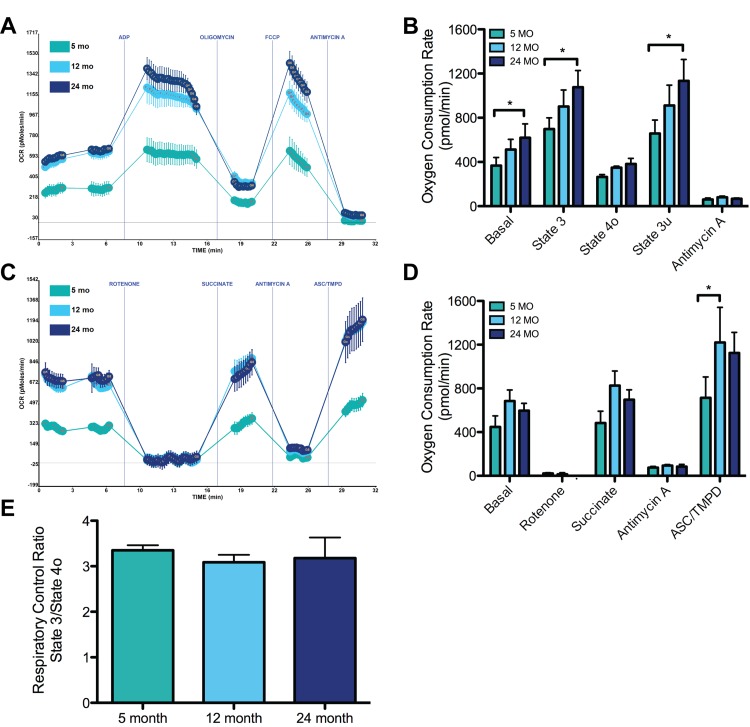
Effects of aging on synaptic mitochondrial bioenergetics Synaptic mitochondria from 5, 12, and 24 month mice were isolated and assayed as described in the Materials and Methods. (**A**) Representative graph output of coupling assay of isolated synaptic mitochondria. Point-to-point oxygen consumption rate (OCR) data are shown with succinate as the substrate followed by addition of ADP, oligomycin, FCCP, and antimycin A. (**B**) Basal (complex II), state 3, state 4o, state 3u respiration. *Significantly (p<0.05) lower in 5 vs. 24 month old animals, n = 5. (**C**) Representative graph output of electron flow assay. Point-to-point OCR data are shown with pyruvate and malate as the substrate followed by the addition of rotenone, succinate, antimycin A, and ASC/TMPD. (**D**) OCR response following rotenone inhibition of complex I, succinate driven complex II, antimycin A inhibition of complex III, and ASC/TMPD driven complex IV. *Significantly (p<0.05) lower in 5 vs. 12 month old animals, n = 3. (**E**) Respiratory Control Ratio (State 3/State 4o) shows no change between 5, 12, and 24 months.

In order to examine the bioenergetic functional correlates of these mitochondrial proteomic changes and of synaptic mitochondria during the aging process, we chose to assess mitochondrial function using the Seahorse XF24 machine, which allows real-time assessment of oxygen consumption rates in cultured cells and isolated mitochondria. Following optimization of the Seahorse bioenergetics assay for isolated synaptic mitochondria (see Methods and Supplemental Figure 2A and 2B), we examined the energetic profiles of mitochondria from 5, 12, and 24 month old mice. To measure mitochondrial coupling between mitochondrial ETC and oxidative phosphorylation (using succinate as the substrate), the rates of basal complex II respiration as well as states 3 (ADP-stimulated respiration), 4o (oligomycin, an ATP synthase inhibitor), and 3u (FCCP, a H^+^ ionophore and uncoupler of oxidative phosphorylation) were consecutively measured. Compared to synaptic mitochondria isolated from 5 month old mice, those from 24 month old mice exhibited a 69% higher rate of complex II driven basal respiration (Figure 4A and 4B). Again at 24 months, when compared to the mitochondria from 5 month old animals, synaptic mitochondria exhibited a 54% increase in ADP-driven state 3 respiration (Figure 4A and 4B). The maximum uncoupled rate from synaptic mitochondria induced by FCCP was also significantly higher in 24 vs. 5 month mice.

Next an electron flow experiment was performed in the presence of FCCP (using pyruvate and malate to drive complex I respiration). We found that compared to 5 months, synaptic mitochondria from 12 and 24 month old mice have an increase in complex I basal respiration (Figure 4C and 4D). Rotenone, an inhibitor of complex I driven respiration, was able to equally inhibit mito-chondria from all ages. Again using succinate revealed that similar to the coupling assay there is increased complex II driven respiration in synaptic mitochondria from 5 month old mice compared those from both 12 and 24 months. Inhibition of complex III with antimycin A decreased the respiratory rate similarly between all ages as expected. Finally, evaluation of complex IV driven respiration with ASC/TMPD revealed a significant increase in the synaptic mitochondria from 12 compared to 5 month old mice suggesting increased complex IV function at 12 months.

However, despite these changes in coupling and electron flow that occur during aging, it is notable that the respiratory control ratio (RCR) values (state3/4o), which is a combined measure of mitochondrial capacity for substrate oxidation, ATP turnover, proton leak and thus is an overall indicator of mitochondrial integrity, were not found to be significantly different between ages in the synaptic mitochondria (Figure 4E). Thus overall function is preserved and the dynamic changes occur at the different ages in synaptic mitochondrial proteomics, these are likely adaptive and do not result in overall differences in mitochondrial energetic capabilities between ages.

### Response to the presence of ROS in aging synaptic mitochondria

**Figure 5:**
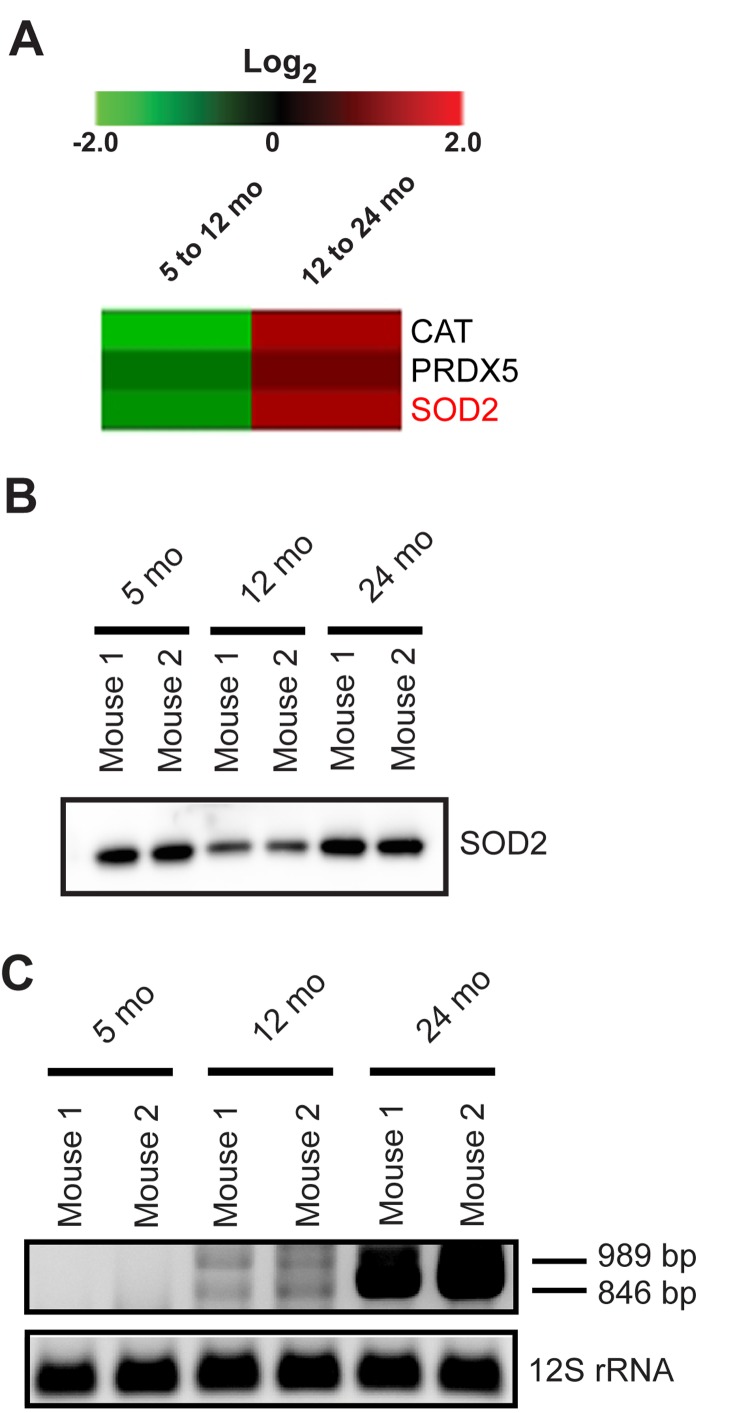
Mitochondrial antioxidant proteins and mtDNA deletions in aging synaptic mitochondria (**A**) Heatmap of the protein expression changes from 5 to 12 months and 12 to 24 months in isolated synaptic mitochondria (log_2_ (12/5 months) and (24/12 months)). SOD2 highlighted in red was verified orthogonally. (**B**) Immunoblot validation of SOD2 protein expression. (**C**) PCR analysis of DNA isolated from synaptic mitochondria revealing accumulation of age-associated mtDNA deletions. PCR product sizes of 989 and 846 bps represent deletions in the mtDNA genome between 9554/13278 and 9088/12956 bps, respectively. PCR of 12S rRNA is used as a control.

Mitochondria are the major source of cellular ROS and ROS production may represent a dysfunction within the mitochondria. However, the idea that mitochondrial ROS are a primary cause of aging has been challenged and several lines of evidence suggest that increased ROS may elicit an important protective response in addition to contributing to age associated damage [23]. As revealed by IPA analysis, several downstream targets of PGC1A identified in our proteomics are antioxidant enzymes, which function to neutralize ROS (Figure 2). The synaptic mitochondrial proteomics show that the expression of the antioxidant enzymes, CAT, PRDX5, and SOD2 decrease from 5 to 12 months but increase from 12 to 24 months, possibly indicating a response to increased ROS in the aged mice (Figure 5A). Orthogonal validation of the changes in SOD2 expression was performed using immunoblotting (Figure 5B). These findings suggest a protective mitochondrial response to an increased presence of ROS at the synapse during aging, which is consistent with previous findings that protein carbonyl content in synaptic mitochondria positively correlates with age [24, 25]. To determine if the observed proteomic changes correlate with increased mitochondrial DNA (mtDNA) damage, we examined the extent of age-associated deletions in synaptic mtDNA. mtDNA mutations were previously thought to be associated with increased oxidative damage, however recent data suggests early mitochondrial replication errors propagate with time [26]. We observed an increased presence of mtDNA deletions within a region that is prone to age-associated damage in synaptic mitochondria from 12 and 24 month old mice (Figure 5C) [27, 28]. The more substantial increase in mtDNA damage from 12 to 24 months also correlates with the observed increased antioxidant enzyme expression. Taken together, these data provide evidence for the presence of a response to increased ROS in aging synaptic mitochondria.

### Proteins involved in mitochondrial dynamics are affected by aging

**Figure 6:**
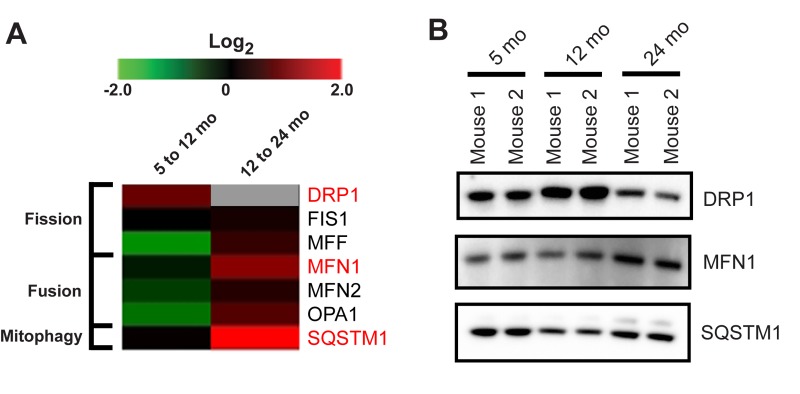
Proteomic changes suggest alterations in mitochondrial dynamics and mitophagy with age (**A**) Heatmap of the protein expression changes from 5 to 12 month and 12 to 24 month in isolated synaptic mitochondria (log2 (12/5 months) and (24/12 months)). Gray box represents value not obtained in our proteomic experiment due to insufficient peptide confidence. Proteins highlighted in red were verified orthogonally. (**B**) Immunoblot validation of protein expression for selected proteins in (**A**).

Mitochondrial dynamics, fission and fusion, are necessary for the maintenance of mitochondrial integrity and play key roles in the function and survival of the cell [29]. Fission and fusion proteins are also regulated during aging (Figure 6). The regulator of mitochondrial fission, dynamin-1-like protein (DRP1), is increased from 5 to 12 months and decreased from 12 to 24 months suggesting a shift to a pro-fusion state in synaptosomes of the aged mice (Figure 6A). Consistent with this finding, the proteins responsible for mediating mitochondrial outer membrane fusion, mitofusin-1 (MFN1) and mitofusin-2 (MFN2) as well as the protein that promotes mitochondrial inner membrane fusion, dynamin-like 120 kDa protein (OPA1), are all decreased from 5 to 12 months and increased from 12 to 24 months, again consistent with a pro-fusion state at 24 months.

The regulation and coordination of the fission and fusion processes are integrally linked with the process of mitophagy. One component of the mitophagy process is the ubiquitin binding protein sequestosome-1 (SQSTM1, also known as p62), which is required for aggregation of damaged mitochondria [30]. While the abundance of SQSTM1 is relatively unchanged in synaptic mitochondria from 5 to 12 months it is highlyupregulated from 12 to 24 months (Figure 6A). These changes were validated by orthogonal immunoblotting (Figure 6B). The recruitment of SQSTM1 to the mitochondria suggests that increased numbers of damaged mitochondria are present in the synaptosomes of aged mice. Moreover, localization of SQSTM1 to mitochondria is associated with the regulation of mitochondrial morphology, genome integrity, and import of TFAM [31]. In fact, increased SQSTM1 protein expression has been shown to directly correlate with levels of mitochondrial TFAM [31]. Our finding of increased SQSTM1 is consistent with the increased synaptic mitochondrial levels of TFAM from 12 to 24 months, as shown above (Figure 2). Together these results suggest a complex interplay between several cellular pathways that work to preserve mitochondrial function with aging.

## DISCUSSION

Because the energetic demands of neurons are heavily dependent on proper mitochondrial function, we sought to investigate the specific mitochondrial proteomic and functional changes corresponding with aging. Several groups have demonstrated, synaptic mitochondria are at high risk for oxidative stress [32] and are specifically more vulnerable to cellular insults such as Ca^2+^ overload [33]. Other lines of evidence also point to mitochondrial dysfunction as a hallmark of aging including mtDNA mutations and the inability of mitochondria to maintain normal function in the face of gradual age related stress and damage [1]. Therefore, in this study we characterized the synaptic mitochondrial proteomic and functional bioenergetic changes that occur with age. Over the course of aging we find that dynamic alterations occur in the synaptic mitochondrial proteome. Somewhat surprisingly there was overall maintenance of mitochondrial function despite direct (mtDNA deletions) and indirect (increased antioxidant protein levels) signs of mitochondrial damage in the aged mice. Aging is not necessarily pathogenic, and in healthy aging, organs, cells and subcellular organelles can respond to gradual age-associated stress, such that adaptive changes to these insults can lead to maintained or even improved outcomes, a concept known as hormesis [34, 35]. The proteomic changes observed likely allow the cell to adapt to functional requirements and counteract age-associated cellular stress and damage enabling maintenance of mitochondrial energetics at the synapse.

Our initial global assessment of the synaptic mitochondrial proteomic changes, based on protein expression, revealed that synaptic mitochondria from 5 and 24 month old mice are slightly more similar than that of 12 months. This change demonstrates a potential proteotypic shift in 24 month old mice back to a 5 month old mitochondrial protein expression pattern. These specific changes in synaptic mitochondrial protein expression may be necessary for the maintenance of ATP production required specifically at the synapse despite age-associated insults. Consistent with this idea, our IPA results predict changes in specific transcriptional regulators responsible for mitochondrial biogenesis, metabolic function, and the antioxidant response. Currently, 2783 proteins are defined by the MitoCarta Inventory of Mammalian Genes to be mitochondrial, almost all are nuclear encoded [36]. In this vein, regulators of nuclear transcription are vital to the regulation of the mitochondrial proteome. Based on our proteomic expression data, the IPA upstream regulators tool predicted two well-characterized transcriptional regulators of nuclear encoded mitochondrial proteins, NRF1 and PCG1A (Figure 2). These two proteins regulate the expression of TFAM, the master regulator of transcription for the mitochondrial genome, which encodes 13 proteins that are all highly essential for mitochondrial function. Based on proteomic data from 5 to 12 months both NRF1 and PGC1A are predicted to be inhibited. Consistent with this prediction, TFAM was decreased from 5 to 12 months (Figure 2C). Alternatively, from 12 to 24 months NRF1 and PGC1A were predicted to be activated and concomitantly TFAM expression was increased. Decreased activation of NRF1 and PGC1A suggest that mitochondrial proteomic upregulation of proteins involved in mitochondrial biogenesis and the antioxidant response do not present until after 12 months. This is consistent with the hypothesis that as aging progresses a threshold is reached where ROS levels can contribute to damage in addition to triggering a protective response. Therefore the upregulation seen from 12 to 24 months may be in response to cellular stress which may promote increased mitochondrial biogenesis [37]. In the later stages of aging, PGC1A, NRF1, and TFAM are upregulated presumably in response to injury to enhance mtDNA levels, OXPHOS activity, and ROS detoxification [37, 38]. Indeed both NRF1 and PGC1A are linked to hormesis [39, 40], and lie upstream of the increase in TFAM. Of note, a PGC1A-independent pathway for the regulation of nuclear encoded mitochondrial proteins including TFAM, via Myc proto-oncogene protein (MYC, also known as c-Myc) and hypoxia-inducible factor 1-alpha (HIF-1α), has recently been reported and suggested to contribute to the age-related decline in mitochondrial function in skeletal muscle [41]. Based on our proteomics data, the IPA upstream regulators tool predicts MYC activity changes that correlate with TFAM expression (Supplemental Figure 4). Our data suggest that these transcriptional changes may provide a compensatory response to aging to maintain mitochondrial function in the context of mitochondria isolated from synaptosomes.

In order to characterize the age-related proteomic changes associated with mitochondrial bioenergetic function, we considered the specific components of the OXPHOS machinery. In the synaptic mitochondria from 5 to 12 months, the OXPHOS subunits from all five complexes decrease and alternatively increase from 12 to 24 month (Figure 3). Generally, decreased activity of OXPHOS proteins is expected with aging and is thought to lead to insufficient energy production and the resulting dysfunction seen in aging mitochondria [42]. Consistent with decreased activity of the OXPHOS complexes, studies in liver and skeletal muscle have shown a decrease in OXPHOS subunit protein expression with aging [43, 44]. However, here we show a dramatic overall increase in the OXPHOS subunit expression from 12 to 24 months (Figure 3). This demonstrates the synaptic mitochondria are distinct in their OXPHOS expression pattern in response to aging and this may be necessary for the maintenance of highly specialized functions over time. Additionally, the observed increased expression of the OXPHOS subunits from 12 to 24 months (Figure 3) is consistent with the hyperfunction theory of aging, an alternative to the damage/maintenance paradigm suggesting that hyperfunction, particularly excess biosynthesis driven by the nutrient-sensitive signaling networks controlling growth and reproduction, during later life causes diverse pathologies whose combinatorial effects lead to aging and death [45-47].

The Seahorse XF analyzer was used to evaluate mitochondrial respiration in isolated synaptic mitochondria to understand the functional mitochondrial changes that are associated with our observed OXPHOS proteomic changes. Others examined the functional changes in synaptic mitochondria associated with aging using different techniques. These studies have demonstrated decreased complex IV and V function in female OF-1 mice at approximately 17 months of age [24, 48], similarly decreases in malate and glutamate driven respiration (complex I) was shown in 3 vs. 28 month old female Fisher Rats [49]. In isolated synaptic mitochondria from 5 vs. 24 month old mice we observed a significant increase in complex II driven basal respiration, state 3, and state 3u respiration (Figure 4). However, RCR values did not differ significantly between ages, indicating that these changes are perhaps adaptive to preserve overall mitochondrial function, as any changes in OXPHOS will alter RCR values [50]. This is in line with other reports from isolated rat heart [51] and kidney [52] mitochondria from aged animals where RCR values do not change. This suggests mitochondrial compensation with aging to maintain normal respiratory function and may be an example of mitochondrial hyperfunction, not declined function during aging. Next we used an electron flow experiment to look at the function of specific ETC complexes (Figure 4C). Based on flux assays it appears that complex II and complex IV substrate utilization is increased in synaptic mitochondria at 12 months. Although this may appear beneficial in the context of aging, complex IV activity requires tight regulation to avoid hyperpolarization of the mitochondrial membrane and increased production of ROS [53]. Despite being initially beneficial, ROS accumulation can lead to detrimental cellular outcomes including mitochondrial dysfunction [54]. Taken together these observed changes in aging synaptic mitochondria are consistent with the hyperfunction theory of aging and have the potential to contribute to age-related pathology [45-47].

ROS are a natural byproduct of respiration and are suggested to be one of the signaling molecules able to induce mitochondrial biogenesis as a mechanism to compensate for ETC defects. Despite the protective signaling role of ROS, as levels surpass the beneficial threshold their presence can promote a maladaptive response. As our proteomic data show, from 12 to 24 months, synaptic mitochondrial levels of the antioxidant enzymes CAT, PRDX5, and SOD2 are increased (Figure 5). This is consistent with previous studies showing increased protein oxidation with age in synaptic mitochondria [24]. In contrast to previous dogma which held that mtDNA mutations result from oxidative damage, most mtDNA mutations in aged cells appear to be caused by early replication errors which propagate with time [1]. Therefore, our finding of increased mtDNA damage suggests that as age-related damage (oxidative or otherwise) continually accumulates, transcriptional changes may invoke a protective response to maintain mitochondrial integrity.

Because the interplay between mitochondrial dynamics and turnover is directly linked to proper mitochondrial function, we characterized the proteomic changes of several key proteins in this process. The fission/fusion process, in addition to mitophagy permits mitochondrial quality control. Synaptic mitochondria show a pattern of increased mitochondrial fission from 5 to 12 months, conversely from 12 to 24 months we observed increased expression of fusion proteins (Figure 6). Since fusion promotes complementation between damaged mitochondria, the shift to a pro-fusion state at 24 months suggests accumulation of synaptic mitochondrial damage. Cellular energy demands and stress can also promote mitochondrial fusion. Consistent with our observation of increased mtDNA damage and upregulation of antioxidant enzymes, the upregulation of synaptic mitochondrial fusion at 24 months may represent a compensatory mechanism. Under these conditions, increased mitochondrial levels of SQSTM1 are most likely not solely for aggregation and transport of damaged mitochondria, but also to recruit TFAM for mtDNA maintenance and biogenesis [31]. According to our IPA analysis and proteomic data, the activity and mitochondrial levels of TFAM change in accordance with SQSTM1 levels in our aged mice.

Here we have highlighted the dynamic synaptic mitochondrial proteomic changes that occur with aging in male C57BL/6 mice. This work suggests changes in transcriptional regulation with age as a compensatory mechanism to alter mitochondrial biogenesis, function, and the antioxidant response. We confirmed an increase in mtDNA damage in aged synaptic mitochondria coinciding with increased TFAM levels when this damage appears to reach a tipping point. Taken together, this work suggests a robust proteomic response is in place to tightly coordinate mitochondrial homeostatic mechanisms. If this response is continued as age-related damage accumulates eventually a breaking point may be reached where this beneficial response becomes counterproductive and can contribute to damage and synaptic dysfunction. However, the observed dynamic changes may also result from a healthy adaptive response to aging (hormesis), which maintains the important functions of mitochondria at the synapse and potentially contributes to aging via mitochondrial hyperfunction. These data provide a framework for further investigation of mitochondrial proteomic changes at the synapse during healthy aging as well as pathological processes that unfortunately increase in prevalence with age.

## METHODS

### Animals

A total of 21 C57BL/6 male mice were obtained from the National Institute on Aging aged rodent colonies: 5 (mature), 12 (old), and 24 (aged) month old ages (7 each) according to annotation standards for age classification from the Neuroscience Information Framework (NIF; http://neuinfo.org) [55]. All protocols were implemented in accordance with NIH guidelines and approved by the Institutional Animal Care and Use Committee at the College of Medicine, University of Nebraska Medical Center. All animals were housed in a controlled room with a constant 12 hour light/dark cycle. Animals were fed standard pellet mouse diet and water *ad libitum*.

### Isolation of synaptic mitochondria for proteomics

Synaptic mitochondria were isolated from 5, 12, and 24 month old C57BL/6 mice using modifications to a method described previously [56]. Following decapitation, brains were rapidly removed and placed in ice-cold isolation medium (IM) containing 225 mM sucrose, 75 mM mannitol, 1 mM EGTA, 5 mM HEPES, and cOmplete Mini, EDTA-free protease inhibitor cocktail (Roche Diagnostics) adjusted to pH 7.4. All homogenization and centrifugation steps were carried out on ice and at 4°C, respectively. Brains were minced and homogenized with 35 strokes in a Dounce homogenizer. The homogenate was then centrifuged at 1,300 x g for 3 min. Supernatant was collected and the pellet was resuspended in IM and centrifuged again at 1,300 x g for 3 min. The pooled supernatants were centrifuged at 21,000 x g for 10 min. This pellet was then resuspended in 15% Percoll and layered on top of a 24% and 40% Percoll gradient (prepared from 100% Percoll solution containing 225 mM sucrose, 75 mM mannitol, 1 mM EGTA, and 5 mM HEPES adjusted to pH 7.4 with HCl). Following centrifugation for 8 min at 30,700 x g the banding near the interface of the upper two layers of the gradient, containing mainly synaptosomes, was collected and diluted in IM. This synaptosomal fraction was then transferred to a nitrogen cavitation vessel (Parr Instrument Company) where the pressure was equilibrated to 900 psi for 15 min followed by depressurization to ATM pressure, which released synaptic mitochondria [56]. This suspension was then added to the top of 24% Percoll and centrifuged for an additional 10 min at 30,700 x g. The pellet containing the synaptic mitochondria was resuspended in IM and centrifuged at 16,700 x g for 10 min. Finally, the pellet was resuspended in IM with fatty acid free BSA followed by centrifugation at 6,900 x g for 10 min. This final pellet, which contains synaptic mitochondria, was further purified using an anti-TOM22 immunomagnetic affinity isolation (Miltenyi Biotech). Resulting mitochondria were lysed in 100 mM Tris-HCl with 4% (w/v) SDS and 0.1M DTT adjusted to pH 7.6. Lysates were incubated at 95°C for 5 min then briefly sonicated. Protein concentrations were determined using a Pierce 660 nm Protein Assay.

### Cell culture and mitochondrial super-SILAC mix preparation

The mouse neural and glial cell lines Neuro-2a, CATH.a, NB41A3, and C8-D1A were obtained from ATCC. All cells were grown in DMEM/F-12 media supplemented with fetal bovine serum, L-glutamine, and penicillin-streptomycin. At 80% confluency, cells were washed twice with PBS, collected, pelleted, and flash frozen in liquid nitrogen followed by storage at −80°C. Cells were lysed and mitochondria were isolated by sequential differential centrifugation using the Mitochondrial Isolation Kit for Cultured Cells (Mitosciences) followed by anti-TOM22 immunomagnetic isolation (Miltenyi Biotec). For stable-isotope labeling by amino acids in cell culture (SILAC) experiments, the four cell lines were SILAC-labeled by culturing in DMEM/F-12 with the heavy isotope-labeled amino acids, (U-^13^C_6_^15^N_4_)-L-arginine (Arg-10) and (U-^13^C_6_)-L-lysine (Lys-6) supplemented with 10% dialyzed fetal bovine serum, SILAC glucose solution, L-glutamine, SILAC phenol red solution, and penicillin-streptomycin. Cells were cultured for at least seven generations in the SILAC media to obtain complete labeling. For the preparation of the mitochondrial super-SILAC mix, equal amounts of each of the four cell line heavy mitochondrial protein lysates were mixed and then combined with unlabeled protein lysates from synaptic mitochondria isolated as described above.

### Protein digestion

Protein was trypsinized using the filter-aided sample preparation technique [19] with a 20 μm filter (Pall Corporation). The resultant peptides were cleaned with an Oasis mixed-mode weak cation exchange cartridge (Waters). Samples were dehydrated with a Savant ISS 110 SpeedVac Concentrator (Thermo Fisher) and resuspended in 40 μl of 0.1% formic acid for LC-MS/MS analysis. Peptides were quantified using a NanoDrop 2000 UV-Vis Spectrophotometer (Thermo Scientific) in conjunction with the Scopes method for protein quantification [57]. The experiment was performed using two technical replicates for each independent biological replicate (*n* = 2).

### LC-MS/MS and data analysis

Mass spectrometry was conducted using an Eksigent ultra nano-HPLC with a cHiPLC system connected to an AB Sciex TripleTOF mass spectrometer equipped with a nanospray configuration. Samples were loaded onto a 200 μm x 6 mm ChromXP C18-CL 3 μm 120 Å trap column (Eksigent), washed with 98:2 LC-MS water with 1% formic acid for 10 minutes and then eluted through a 200 μm x 15 cm ChromXP C18-CL 3 μm 120 Å analytical column (Eksigent) at a rate of 300 nl/min with a linear gradient of acetonitrile from 0-60% over the course of 200 min. The instrument was calibrated using 25 fmol of beta galactosidase standards. Data obtained from the TripleTOF was analyzed with Protein Pilot version 4.5 (Paragon Algorithm: 4.5.0.0, 1654) software to generate peptide and protein lists. Using the Paragon method [20], the peak lists were compared against the Uniprot mouse database. Ratios of the amount of heavy-to-light (H:L) (labeled-to-unlabeled) peptide were generated. The search parameters were set as a maximum of two missed cleavages, carbamidomethyl (C) as fixed modification, N-acetyl (protein) and oxidation (M) as variable modifications, top 6 MS/MS peaks per 100 Da, and MS/MS mass tolerance of 0.5 Da. Exclusion criteria to remove proteins from analysis were as follows: FDR of 0.05 for both peptides and proteins, peptides must contain at least 6 amino acids, contaminants as identified through the database search and proteins identified as being in the reverse database. The additional cutoff values of Unused ProtScore ≥ 1.3 and number of peptides ≥ 1 were applied to the data.

### Bioinformatic analysis

Hierarchical clustering of proteins was performed using Partek Genomics Suite 6.6 version 6.12.0713 software (Partek Inc.). MitoMiner (MRC: Mitochondrial Biology Unit; mitominer.mrc-mbu.cam.ac.uk) was used to annotate our identified and quantified proteins as mitochondrial. The Ingenuity Pathway Analysis (IPA: Ingenuity Systems; http://www.ingenutiy.com/) Upstream Regulator and Downstream Effects Analysis tool was used to predict alterations in the activity of mitochondrial transcriptional regulators based on our uploaded protein expression data.

### Immunoblotting

Synaptic mitochondrial lysates were prepared as described above from mouse brain tissue. Immunoblotting was completed as previously described [58]. Briefly, equal amounts of protein (10 μg) were loaded onto 4-12% Bis-Tris gels, transferred to nitrocellulose membranes, blocked, and incubated with the following antibodies overnight at 4°C: the oxidative phosphorylation (OXPHOS) panel (1:5000) (MS604; Mitoscences), SOD2 (1:10,000) (ab16956; Abcam), VDAC1 (1:10,000) (4661; Cell Signaling), ATP5H (1:10,000) (MS504; Mitosciences), MFN1 (1:2,000) (ab57602; Abcam), TFAM (1:4,000) (LS-C30495; LifeSpan Biosciences), DRP1 (1:2000) (D8H5, Cell Signaling), and SQSTM1 (1:8000) (PM045, MBL). The OXPHOS antibody panel is a mix of antibodies that include: NDUFB8, SDHB, UQCRC2, MTCO1, and ATP5A1. Since commonly used mitochondrial protein loading controls (VDAC1 and GAPDH) change during aging according to our proteomics and under different conditions [59, 60], we performed Coomassie staining to confirm equal protein loading (Supplemental Figure 1). Ponceau staining was done to confirm equal protein loading for each membrane. Chemiluminescent bands were visualized with an Image Station 4000MM Pro and analyzed using Carestream Molecular Imaging software (both from Carestream Health, Inc.).

### Isolation and bioenergetic analysis of isolated brain mitochondria

Synaptic mitochondria were isolated from 5, 12, and 24 month old mice as described above in methods section 2.2 with the following modifications. The mouse brains were homogenized in mitochondrial isolation buffer (MSHE+BSA): 70 mM sucrose, 210 mM mannitol, 5 mM HEPES, 1 mM EGTA and 0.5% (w/v) fatty acid free BSA (pH 7.2) using only 10 strokes in a Dounce homogenizer. Following the Percoll centrifugation, the final centrifugation steps were performed at 8,000 x g for 10 min. The anti-TOM22 immunomagnetic affinity isolation purification step was not performed. Final pellets for bioenergetic analysis were resuspended in a minimal volume of MSHE+BSA. Total mitochondrial concentrations were determined using the BCA method. Isolated mitochondria were used immediately for analysis. In order to optimize the Seahorse bioenergetics assay for our established mitochondrial isolation protocol, synaptic mitochondria at 2, 5, 10, and 20 μg were loaded onto the Seahorse XF24 using a modified protocol of Rogers et al. [61] (Supplemental Figure 2A and 2B). Optimization of synaptic mitochondria demonstrated that mitochondrial amounts greater than 10 μg per well have a non-linear state 3 rate (Supplemental Figure 2A), therefore we used 10 μg of mitochondria for subsequent experiments. The Seahorse XF24 Flux Analyzer (Seahorse) was equilibrated to 37°C overnight and a modified protocol was used based on previous work in mouse liver mitochondria [61]. The isolated synaptic mitochondria (10 μg) were plated in V7-PS XF24 cell culture microplates in a volume of 50 μl mitochondrial assay solution (MAS) containing 70 mM sucrose, 220 mM mannitol, 10 mM KH_2_PO_4_, 5 mM MgCl_2_, 2 mM HEPES, 1 mM EGTA and 0.2% fatty-acid free BSA with 10 mM succinate and 2 μM rotenone as substrate for the coupling assay or 10 mM pyruvate, 2 mM malate, and 4 μM FCCP for the electron flow experiment. After centrifugation for 15 min at 2,000 x g to attach mitochondria, 450 μl of MAS (containing substrate) was added to each well, and the plate was incubated at 37°C for 8 min to equilibrate temperature. The final concentrations of additions to the wells were 4 mM ADP, 2.5 μg/ml oligomycin, 4 μM FCCP, and 4 μM antimycin A for the coupling assay and 2 μM rotenone, 10 mM succinate, 4 μM antimycin A, and 10 mM ascorbate with 100 μM TMPD for the electron flow assay. The coupling and electron flow assays were run in 3-4 technical replicate wells for each independent biological replicate (coupling assay, *n* = 5; electron flow assay, *n* = 3). XF24 data was calculated using the algorithm previously described and used by the Seahorse software package [62]. Statistical analysis was conducted in PRISM (GraphPad Software) using one-way ANOVA and post-test with Tukey's multiple comparison tests.

### PCR mtDel assay

Oligonucleotide primers were synthesized (Eurofins MWG Operon) to anneal to mtDNA segments flanking three direct repeats in the regions 8884-13357 [63]. mtDNA was prepared from the synaptic mitochondrial fractions using the QIAamp DNA Micro Kit (Qiagen); the oligonucleotide outer primers, 5'-TAATTCAAGCCTACGTATTC-3' (forward) and 5'-GGGATGTTTTTAGGCTTAGG-3' (reverse), and the oligonucleotide nested primers, 5'-CAAGTCCATGACCATTAACTGG-3' (forward) and 5'-GATTTTATGGGTGTAATGCG-3' (reverse) were used for the mtDNA deletion PCR reaction. As undeleted mtDNA controls in each sample, the mtDNA segment (471-670) encoding 12S rRNA was also amplified using 5'-GACAGCTAAGACCCAAACTG-3' (forward) and 5'-TTAGCAAGAGATGGTGAGGT-3' (reverse) primers. PCR conditions were initial denaturation at 94°C for 4 min, followed by 30 cycles of denaturation, annealing and extension at 94°C for 20 sec, 55°C for 20 sec and 72°C for 20 sec, respectively, and final extension at 72°C for 4 min; the FastStart High Fidelity PCR System, dNTPack (Roche) was used. The outer primers were used for the first 30 cycles as described above with 20 ng mtDNA template, then 2 μl of the first reaction was transferred to the nested PCR for the second 30 cycles. Resulting reactions were visualized by agarose gel electrophoresis.

## SUPPLEMENTAL FIGURES


