# Supplementary material for: Aging synaptic mitochondria exhibit dynamic proteomic changes while maintaining bioenergetic function
Source: Aging (Albany NY). 2014 May 5;6(4):320–34. doi: 10.18632/aging.100657 (PMC4032798; doi:10.18632/aging.100657)
Supplement: Supplementary file 1 [file aging-06-320-s001.pdf]

(VDAC) is involved in apoptosis of cell lines carrying the mitochondrial DNA mutation. BMC Med Genet. 2009; 10:114.
**60.** Ferguson RE, Carroll HP, Harris A, Maher ER, Selby PJ and Banks RE. Housekeeping proteins: a preliminary study illustrating some limitations as useful references in protein expression studies. Proteomics. 2005; 5:566-571.
**61.** Rogers GW, Brand MD, Petrosyan S, Ashok D, Elorza AA, Ferrick DA and Murphy AN. High throughput microplate respiratory measurements using minimal quantities of isolated mitochondria. PLoS One. 2011; 6:e21746.
**62.** Gerencser AA, Neilson A, Choi SW, Edman U, Yadava N, Oh RJ, Ferrick DA, Nicholls DG and Brand MD. Quantitative microplate-based respirometry with correction for oxygen diffusion. Anal Chem. 2009; 81:6868-6878.
**63.** Wang E, Wong A and Cortopassi G. The rate of mitochondrial mutagenesis is faster in mice than humans. Mutat Res. 1997; 377:157-166.

## SUPPLEMENTAL DATA

Please browse full text version to see the Supplemental Tables of this manuscript. Supplementary File 1. Complete list of the 898 common identified and quantified proteins. Supplementary File 2. Complete MitoMiner annotation output.

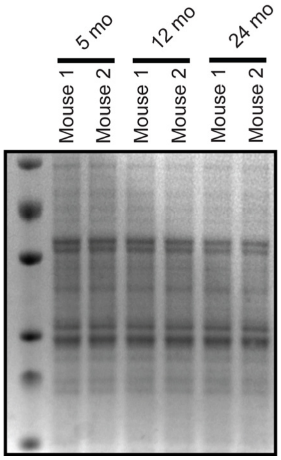


**Supplemental Figure 1. Confirmation of equal loading for immunoblots.** Coomassie staining of 10 μg total protein for each synaptic mitochondrial sample loaded for electrophoresis.

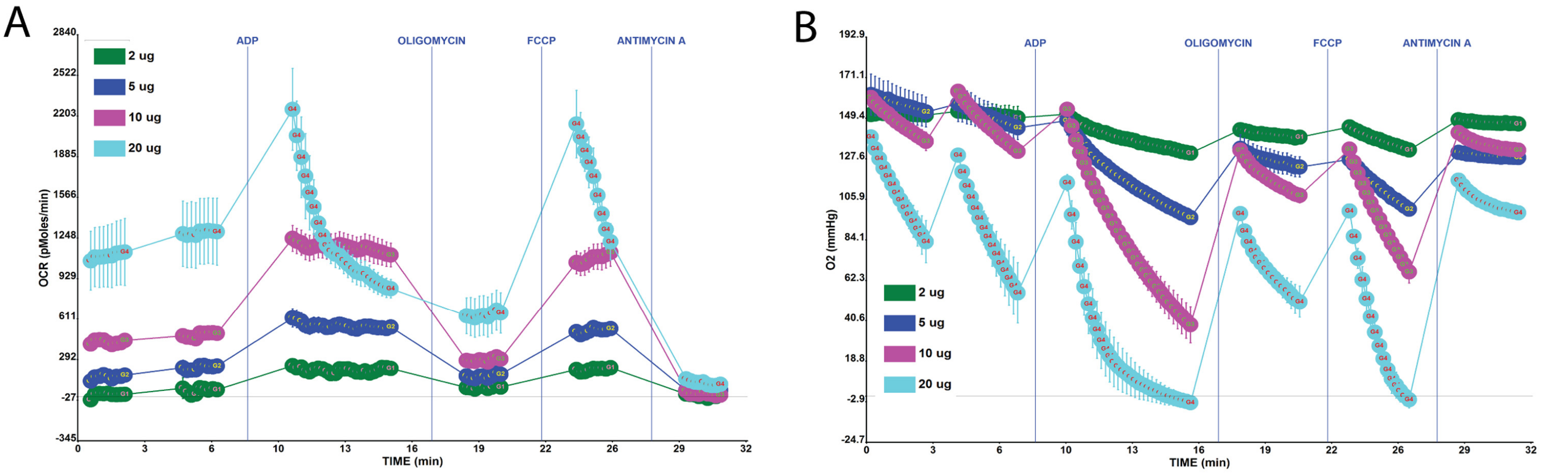


**Supplemental Figure 2. Optimization of Seahorse XF24 with isolated mouse synaptic mitochondria.** (**A**) OCR is shown for coupling assay with 2, 5, 10 and 20 µg of synaptic mitochondria. ADP, FCCP, oligomycin, and antimycin A were added as described in the Materials and Methods. (**B**) Absolute levels of $O_2$ in the wells are shown from the same assay in (**A**).

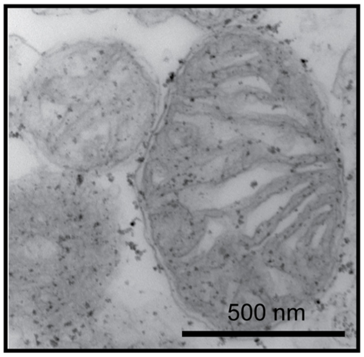


**Supplemental Figure 3. Electron microscopy of the pure isolated synaptic mitochondria.** Electron dense spots indicate the TOM22 magnetic beads, which remain after the immunomagnetic affinity purification step. Magnification shown is 52,000x.

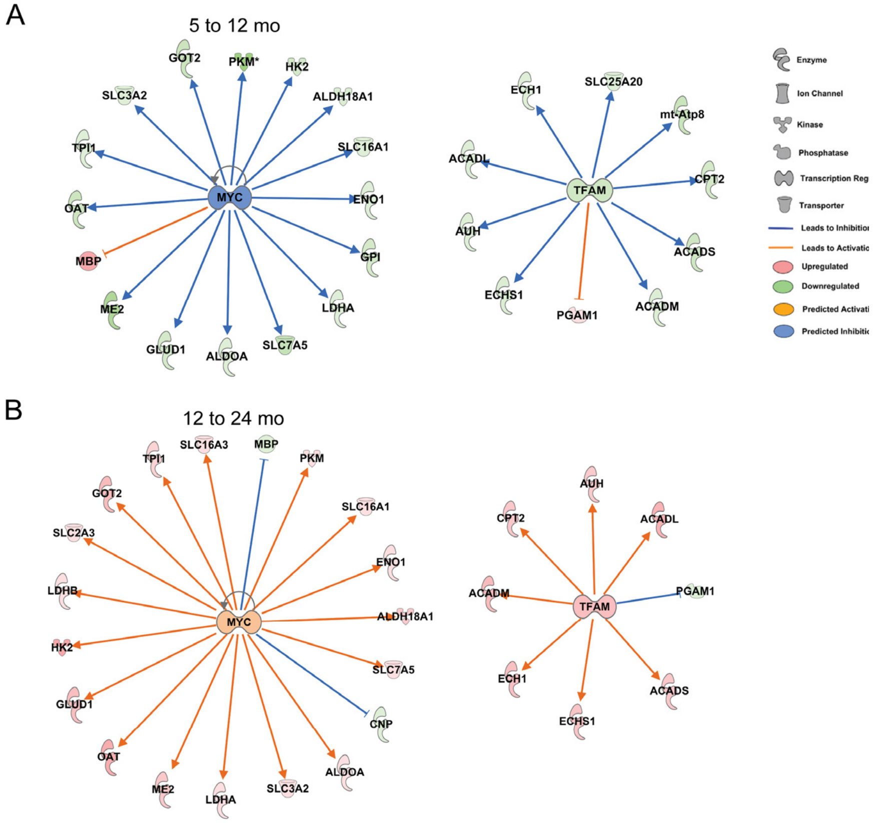


**Supplemental Figure 4. Proteomics predicts changes in a PGC1A-independent mitochondrial transcriptional regulatory pathway with aging.** IPA generated MYC and TFAM upstream regulator networks overlaid with our proteomic expression data for synaptic mitochondria from (**A**) 5 to 12 month and (**B**) 12 to 24 month old mice. MYC and TFAM activity was predicted based on proteomic expression data. All proteins shown (except MYC) were found in the proteomic analysis.
